# Characterization of CetA and CetB, a bipartite energy taxis system in *Campylobacter jejuni*

**DOI:** 10.1111/j.1365-2958.2008.06357.x

**Published:** 2008-07-23

**Authors:** Kathryn T Elliott, Victor J DiRita

**Affiliations:** 1 Department of Microbiology and Immunology, University of MichiganAnn Arbor, MI 48109, USA; 2 Unit for Laboratory Animal Medicine, University of MichiganAnn Arbor, MI 48109, USA

## Abstract

The energy taxis receptor Aer, in *Escherichia coli*, senses changes in the redox state of the electron transport system via an flavin adenine dinucleotide cofactor bound to a PAS domain. The PAS domain (a sensory domain named after three proteins Per, ARNT and Sim, where it was first identified) is thought to interact directly with the Aer HAMP domain to transmit this signal to the highly conserved domain (HCD) found in chemotaxis receptors. An apparent energy taxis system in *Campylobacter jejuni* is composed of two proteins, CetA and CetB, that have the domains of Aer divided between them. CetB has a PAS domain, while CetA has a predicted transmembrane region, HAMP domain and the HCD. In this study, we examined the expression of *cetA* and *cetB* and the biochemical properties of the proteins they encode. *cetA* and *cetB* are co-transcribed independently of the flagellar regulon. CetA has two transmembrane helices in a helical hairpin while CetB is a peripheral membrane protein tightly associated with the membrane. CetB levels are CetA dependent. Additionally, we demonstrated that both CetA and CetB participate in complexes, including a likely CetB dimer and a complex that may include both CetA and CetB. This study provides a foundation for further characterization of signal transduction mechanisms within CetA/CetB.

## Introduction

Motile bacteria alter the direction in which they swim based on changes in the local environment. These changes can be sensed directly in classical chemotaxis, where changes in the local concentration of a stimulus (i.e. an amino acid or sugar) are sensed in a metabolism-independent fashion, often by transmembrane methyl-accepting chemotaxis proteins (MCPs). Changes in the local environment can also be sensed indirectly by monitoring energy-generating processes within the cell. In this behaviour, termed energy taxis, receptors sense changes in the redox state of components of the electron transport system (ETS) or in the closely coupled proton motive force (Taylor and Zhulin, 1998). Energy taxis behaviours include some forms of aerotaxis, phototaxis, taxis to electron acceptors and even chemotaxis in those cases where the bacteria sense chemicals based on changes in energy generation resulting from their metabolism (Taylor and Zhulin, 1998; Taylor *et al*., 1999; Alexandre *et al*., 2004).

Energy taxis receptors and their signal transduction mechanisms have been well-characterized in *Escherichia coli*. *E. coli* contains two energy taxis receptors: Tsr, a classic serine-responsive MCP that also senses changes in the proton motive force, and Aer, which senses changes in the redox state of element(s) of the ETS (Rebbapragada *et al*., 1997). Aer has been suggested to sense these changes via the redox state of an flavin adenine dinucleotide cofactor bound to the N-terminal PAS domain (a sensory domain named after three proteins Per, ARNT and Sim, where it was first identified) (Taylor, 2007). This signal is thought to be transmitted to the HAMP domain of Aer (named for its presence in histidine kinases, adenylyl cyclases, MCPs and phosphatases) (Aravind and Ponting, 1999) by a direct PAS–HAMP interaction (Taylor, 2007). Finally, the HAMP domain relays the signal to the highly conserved domain (HCD) (named for its prevalence in MCPs) (Taylor, 2007). Aer also possesses two transmembrane domains with a small intervening periplasmic loop, but there is, as yet, no evidence for the involvement of this region in signal transduction (Amin *et al*., 2006). This differs from classical MCPs which are thought to transmit signals sensed by a periplasmic domain to the HAMP and HCD domains by a shift in their transmembrane helices (Chervitz and Falke, 1996; Moukhametzianov *et al*., 2006).

Flagellar motility plays a vital role in the pathogenicity of *Campylobacter jejuni*, one of the most common causes of gastroenteritis in the United States, as well as in its colonization of livestock reservoirs, most commonly poultry (Guerry, 2007; Young *et al*., 2007). A transposon screen of mutants with defects in motility identified insertions in *cetA* and *cetB*, adjacent genes on the *C. jejuni* genome that encode proteins representing a variation on the domain arrangement found in Aer (Hendrixson *et al*., 2001). CetB contains a predicted PAS domain and no other functional domains. CetA is predicted to contain a transmembrane region, a HAMP domain and the HCD. Mutants lacking *cetA* or *cetB* are deficient in energy taxis (Hendrixson *et al*., 2001). These studies led to the hypothesis that CetA and CetB interact to transduce an energy taxis signal via a similar mechanism as that proposed for the single protein Aer. Specifically, we predict that CetB interacts with the HAMP domain of CetA, as is suggested of the PAS and HAMP domains of Aer. However, significant divergence between the HAMP domains of Aer and CetA suggests that the molecular nature of these interactions likely differ (K.T. Elliott, I.B. Zhulin, J.A. Stuckey, V.J. DiRita, in revision). We have determined that CetA and CetB define a new family of HAMP/PAS containing proteins, with pairs of similar proteins identified in 22 species thus far (K.T. Elliott, I.B. Zhulin, J.A. Stuckey, V.J. DiRita, in revision).

In this study, we initiated molecular and biochemical characterization of CetA and CetB, testing predictions about their transcription, topology, localization and interaction. Our studies show that *cetA* and *cetB* are co-transcribed independently of the flagellar regulon. Further, CetA and CetB are both membrane-associated: CetA by two transmembrane helices in a helical hairpin; CetB in a peripheral manner, likely via protein–protein interactions. In addition, we uncovered evidence of a likely protein–protein interaction between CetA and CetB, including low levels of CetB in the absence of CetA, and the existence of high molecular weight complexes that appear to include both proteins.

## Results

### *cetA* and *cetB* are co-transcribed independently of the flagellar regulon, and CetB levels are CetA-dependent

**Fig. 1:**
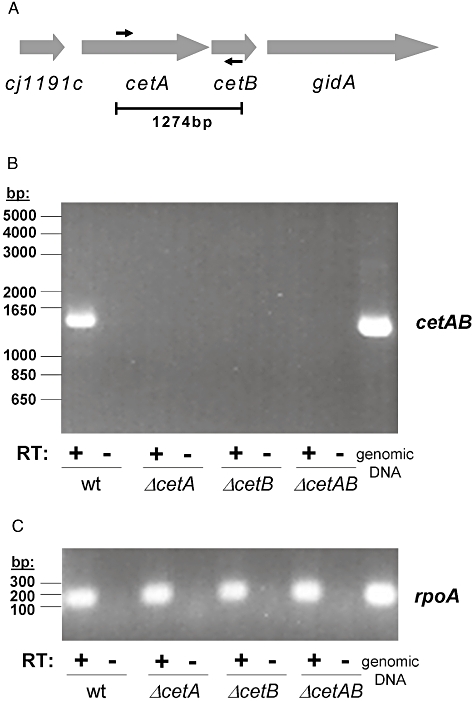
RT-PCR analysis of *cetAB* locus. A. Location of primers within *cetA* and *cetB* used to determine if both genes are present on one transcript. Expected PCR product size is given. B. RT-PCR results using primers shown in (A). C. RT-PCR results using primers within *rpoA* that result in an 180 bp product. In both (B) and (C), results from RT-PCR using RNA from wild type, Δ*cetA*, Δ*cetB* and Δ*cetAB* are shown. Control reactions were performed in which reverse transcriptase (RT) was omitted from the cDNA synthesis reaction, in order to rule out the presence of contaminating DNA. As a positive control, the PCR products resulting from use of genomic DNA as the template are also shown.

Our hypothesis that CetA and CetB interact to transduce an energy taxis signal is based in part on the fact that they are encoded by adjacent genes on the *C. jejuni* chromosome. As there are 17 bp between the *cetA* and *cetB* genes, we expected that they would be co-transcribed. We tested this prediction by performing reverse transcription polymerase chain reaction (RT-PCR) using one primer within each gene (Fig. 1A). If both *cetA* and *cetB* are present on the same transcript, then a single product spanning both genes would arise from these primers. This predicted product was present when the wild-type RNA was used as the template for RT-PCR, but not when RNA from the Δ*cetA*, Δ*cetB* or Δ*cetAB* mutants was used or when reverse transcriptase was omitted from the reaction (Fig. 1B). A product of the same size was also visible when genomic DNA was used for the PCR template. As a control, we performed RT-PCR with primers within the *rpoA* gene, which encodes the housekeeping sigma factor, σ^70^. The *rpoA* RT-PCR product was evident in all RT samples (Fig. 1C). These data support our hypothesis that *cetA* and *cetB* are co-transcribed.

**Fig. 2:**
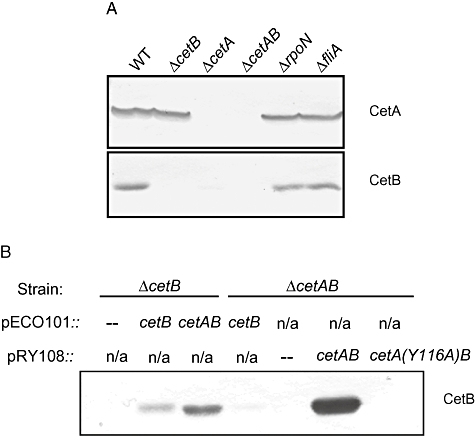
Expression of CetA and CetB in various genetic backgrounds. A. Whole-cell extracts were prepared from wild type, Δ*cetB*, Δ*cetA*, Δ*cetAB*, Δ*rpoN* and Δ*fliA*. These were separated by 12.5% SDS-PAGE and CetA and CetB detected by immunoblotting. B. Whole cell extracts were prepared from Δ*cetB* and Δ*cetAB* with pECO101, pECO101*::cetB*, pECO101*::cetAB*, pRY108, pRY108*::cetAB* or pRY108*::cetA*(*Y116A)cetB*. These samples were separated by 12.5% SDS-PAGE and CetA and CetB detected by immunoblotting.

*Campylobacter jejuni* has only three known sigma factors identified within its genome: σ^70^, σ^54^ (encoded by *rpoN*) and σ^28^ (encoded by *fliA*). The latter two sigma factors are required for the flagellar transcriptional cascade in *C. jejuni* (Hendrixson and DiRita, 2003). Levels of CetA and CetB were unaffected in strains lacking σ^54^ or σ^28^, indicating that *cetA* and *cetB* are likely expressed in a σ^70^-dependent fashion (Fig. 2A).

Western blots also demonstrated that CetB levels are at or below our limit of detection in the Δ*cetA* mutant (Fig. 2A). This is true whether CetB is expressed from the chromosome (Fig. 2A) or from a plasmid under the control of a constitutive promoter (Fig. 2B), indicating that the low level of CetB expression in the Δ*cetA* mutant is not due to a polar effect of this in-frame deletion. Additionally, when both CetA and CetB are expressed from a plasmid bearing their native promoter, but CetA is rendered unstable by a HAMP domain point mutation (Y116A), CetB levels are also quite low (Fig. 2B). The loss of stability of one protein in the absence of another is a frequent indication of a protein–protein interaction. Our data are consistent with CetB stability being CetA-dependent; however, we cannot rule out a potential effect of CetA on CetB translation. Cj1191c, an apparent CetB paralogue encoded by the gene upstream of *cetA*, exhibits no role in energy taxis (Hendrixson *et al*., 2001). Unlike those of CetB, Cj1191c levels are not CetA-dependent (data not shown).

### CetA has two transmembrane domains in a helical hairpin

**Fig. 3:**
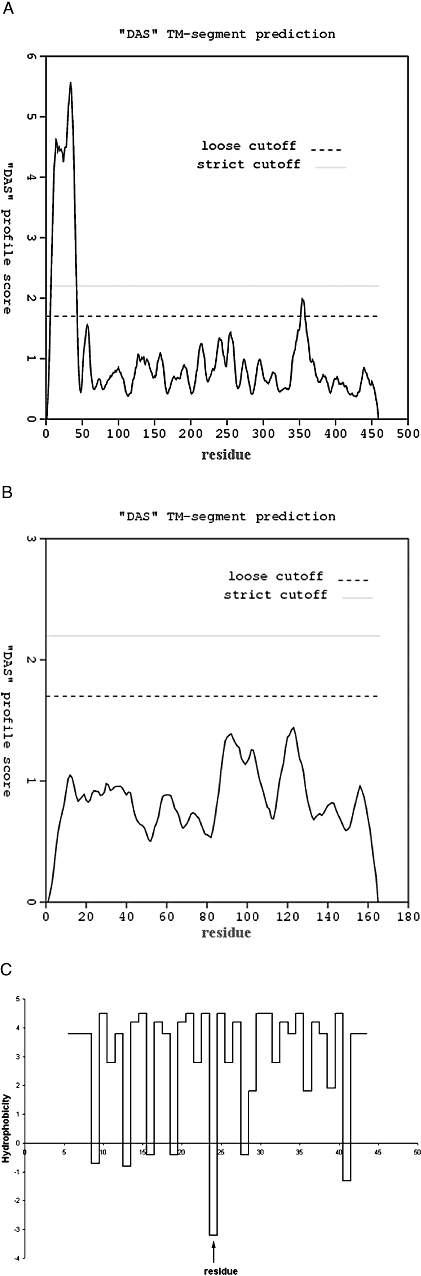
Prediction of transmembrane regions for CetA and CetB. A. DAS analysis results for CetA. B. DAS analysis results for CetB. C. Hydrophobicity (Kyte–Doolittle values) of each residue within the DAS-predicted transmembrane region of CetA. Arrow indicates position of His-24.

We used the DAS (dense alignment surface) algorithm (Cserzo *et al.*, 1997) to predict whether CetA and/or CetB possess transmembrane domains. According to this analysis, CetA has a transmembrane region of 36–38 amino acids in length from residues 6–43 or 7–42, depending on the cut-off used (Fig. 3A).

The DAS analysis indicates that CetB does not have any transmembrane domains (Fig. 3B). As the LipoP programme indicates that CetB also does not possess a signal sequence (data not shown), we predict that CetB is located in the *C. jejuni* cytoplasm.

A dip in the DAS profile score is apparent at about the mid-point of the predicted transmembrane region in CetA. Closer examination of the hydrophobicity of each residue showed a strongly hydrophilic residue near the mid-point of this region (arrow, Fig. 3C). This residue is a histidine (His-24) which is known to induce turns in transmembrane helices, giving rise to helical hairpins (Monne *et al*., 1999a,b). Additionally, the predicted transmembrane region is flanked on either side by positively charged residues (K3, R44, H46, K47). Such residues near transmembrane helices have been proposed to act like the flukes of an anchor to moor the protein into the membrane, with the positive residues residing on the cytoplasmic surface of the protein (Boyd and Beckwith, 1989). These observations led us to hypothesize that CetA may contain two transmembrane helices in a helical hairpin confirmation, as opposed to the single transmembrane helix predicted by DAS analysis.

To differentiate between the single transmembrane helix predicted by the DAS algorithm and our prediction that CetA has two transmembrane helices, we performed topology analysis using *phoA* and *lacZ* fusions. *phoA* encodes alkaline phosphatase, which is active in the periplasm and inactive in the cytoplasm. *lacZ* encodes β-galactosidase, an enzyme that is active in the cytoplasm and too bulky to be transported to the periplasm. Fusion of β-galactosidase to periplasmic regions of a protein leads to the embedding of the fusion in the membrane, resulting in improper folding and a loss of enzymatic activity (Froshauer *et al*., 1988). By comparing alkaline phosphatase and β-galactosidase activities resulting from fusions at various locations within a protein, we can develop a good prediction of the topology of that protein (Silhavy and Beckwith, 1985; Manoil and Beckwith, 1986; Manoil, 1990).

**Fig. 4:**
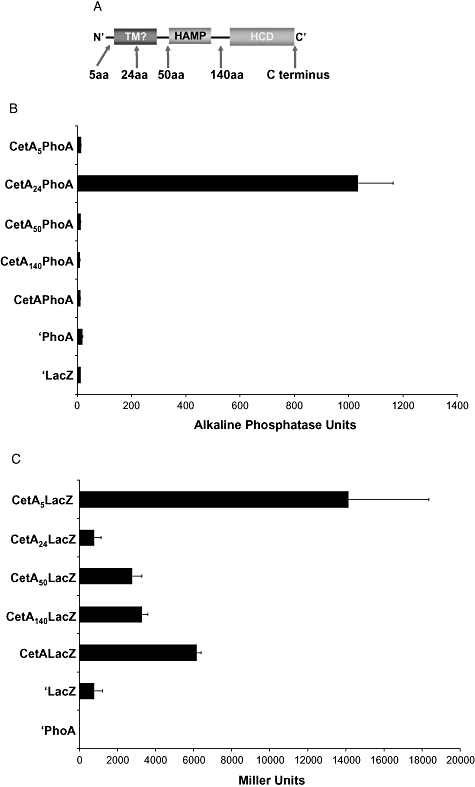
location and activities of LacZ and PhoA fusions to CetA. A. Locations of PhoA and LacZ fusions to truncated or full-length CetA are indicated. LacZ or PhoA was fused C-terminally to the truncated or full-length protein. B. Alkaline phosphatase activity of PhoA fusions. C. β-Galactosidase activity of LacZ fusions. In both (B) and (C), ‘PhoA’ and ‘LacZ’ indicate the empty vectors pTrcphoA and pTrcLacZ respectively.

We made *phoA* and *lacZ* fusions such that alkaline phosphatase or β-galactosidase would be fused C-terminally to full-length CetA or to CetA that was truncated at residue 5, 24, 50 or 140 (Fig. 4A). These fusions were expressed in an *E. coli* strain lacking *lacZ* and *phoA* and assayed for alkaline phosphatase and β-galactosidase activity. The only alkaline phosphatase fusion construct with significant activity was that at His-24 of CetA (Fig. 4B). The β-galactosidase fusion at this location (His-24) was also the fusion with the lowest β-galactosidase activity (Fig. 4C). This fusion did have β-galactosidase activity above background levels, however. Work in other laboratories has indicated that β-galactosidase fusions to periplasmic regions, which do not lead to translocation of the fusion but rather embed the protein in the membrane, can sometimes lead to degradation of the fusion and release of native β-galactosidase, giving rise to activity (Georgiou *et al*., 1988; Gott and Boos, 1988). These studies caution that use of β-galactosidase fusions must be complemented by an alternative topological probe, such as alkaline phosphatase, which is likely a more reliable indicator of subcellular localization. Together, our results indicate that His-24 is accessible to the periplasm, whereas all of the other fusion locations are found in the cytoplasm. These data support our prediction that CetA has two transmembrane helices in a helical hairpin.

### CetA is an integral membrane protein and CetB is a peripheral membrane protein

**Fig. 5:**
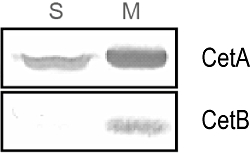
Subcellular fractionation of CetA and CetB. Whole cell lysates were separated into soluble and membrane fractions by ultracentrifugation. These fractions were separated by 12.5% SDS-PAGE. CetA and CetB were detected by immunoblotting. Membrane fractions contained < 10% of the isocitrate dehydrogenase specific activity.

The above *phoA*/*lacZ* fusion experiments were performed in *E. coli*. We sought to determine the localization of CetA and CetB in *C. jejuni*. To do so, we prepared and analysed subcellular fractions for localization of CetA and CetB. Wild-type *C. jejuni* was lysed as described in Materials and Methods. Soluble and membrane-associated proteins were then separated by ultracentrifugation. These samples were analysed for the presence of CetA and CetB by Western blot. While some CetA is detectable in the soluble fraction, the majority of both CetA and CetB are in the membrane fraction (Fig. 5). Less than 10% of the isocitrate dehydrogenase specific activity (Myers and Kelly, 2005) was found in membrane fractions during these experiments. The presence of a minority of soluble CetA could be due to incomplete fractionation or the presence of newly synthesized protein that has not yet localized to the membrane (Crawford *et al*., 2003).

As DAS analysis indicated that CetB does not have a transmembrane region, the presence of CetB in the membrane fraction suggests that CetB is a peripheral membrane protein, associated with the membrane by protein–protein interactions or by direct interaction with the membrane. In order to determine the nature of the association of CetB with the membrane, we performed membrane extraction experiments. In these experiments, a *C. jejuni* strain was used that expressed a FLAG-tagged CtsP protein, which had been previously characterized by our lab as a peripheral membrane protein (R.S. Wiesner and V.J. DiRita, in preparation). The bacteria were lysed and separated into soluble and membrane fractions as described above. The membrane fraction was washed three times in 10 mM HEPES (pH 7.4) prior to treatment with urea, NaCl or buffer alone. Urea denatures proteins and disrupts protein complexes, thereby releasing peripheral membrane proteins (Gilmore and Blobel, 1985; Borel and Simon, 1996). High-salt treatment weakens ionic interactions between peripheral membrane proteins and other membrane proteins or the polar head groups of the lipid bilayer (Kretzschmar *et al*., 1996; Hugle *et al*., 2001). Integral membrane proteins should remain insoluble following treatment with urea or high salt. Peripheral membrane proteins may be soluble following urea and/or high-salt treatment depending on the nature and strength of their membrane association.

**Fig. 6:**
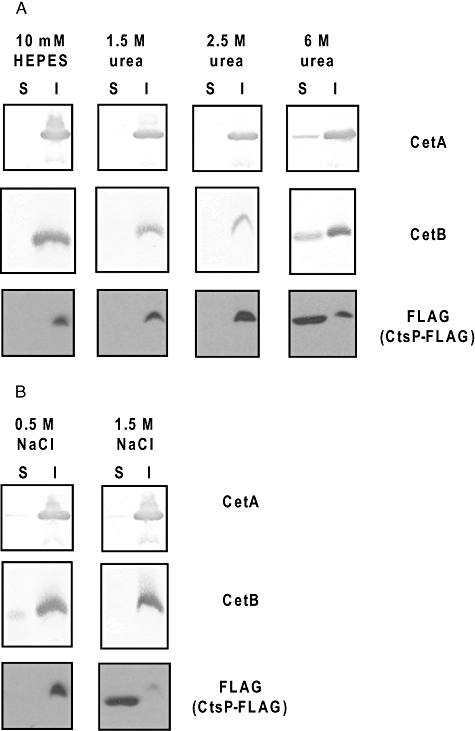
Membrane extractability of CetA and CetB. A. Membrane extractability in buffer alone or in urea. B. Membrane extractability in NaCl. Membrane fractions following subcellular fractionation of wild-type cells expressing CtsP-FLAG (a peripheral membrane protein) were washed in 10 mM HEPES, then treated with buffer alone (10 mM HEPES) or buffer containing the indicated concentrations of urea or NaCl. Soluble and insoluble proteins were separated by ultracentrifugation. Samples were separated on 12.5% SDS-PAGE and probed for the presence of CetA, CetB and CtsP-FLAG by immunoblotting.

After these treatments, the soluble and insoluble proteins were separated by ultracentrifugation and probed for the presence of CetA, CetB and CtsP-FLAG. CtsP was solubilized in 6 M urea (Fig. 6A), while CetB was only partially solubilized in 6 M urea, and CetA remained insoluble following this treatment (Fig. 6A). CtsP was soluble in 1.5 M NaCl, but both CetA and CetB remained insoluble following high-salt treatment (Fig. 6B). CetA and CetB are both soluble following treatment with 0.15% Triton X-100 (data not shown), indicating that they are soluble when the membrane itself is disrupted. From these results, we conclude that CetA is an integral membrane protein and CetB is a peripheral membrane protein. The partial release of CetB from the membrane following treatment with urea, but not high salt, indicates that CetB has an avid association with the membrane, possibly as a result of protein–protein interactions.

### CetA and CetB associate in larger complexes

In order to test further whether CetA and CetB interact with one another and/or other proteins, we performed *in vivo* cross-linking experiments. Cells were treated with 2.5 mM of the membrane permeable primary amine cross-linker DSP in DMSO or with DMSO alone. DSP was inactivated by addition of 50 mM Tris pH 8.0, and these samples were analysed by non-reducing SDS-PAGE and probed for CetA or CetB by Western blot.

**Fig. 7:**
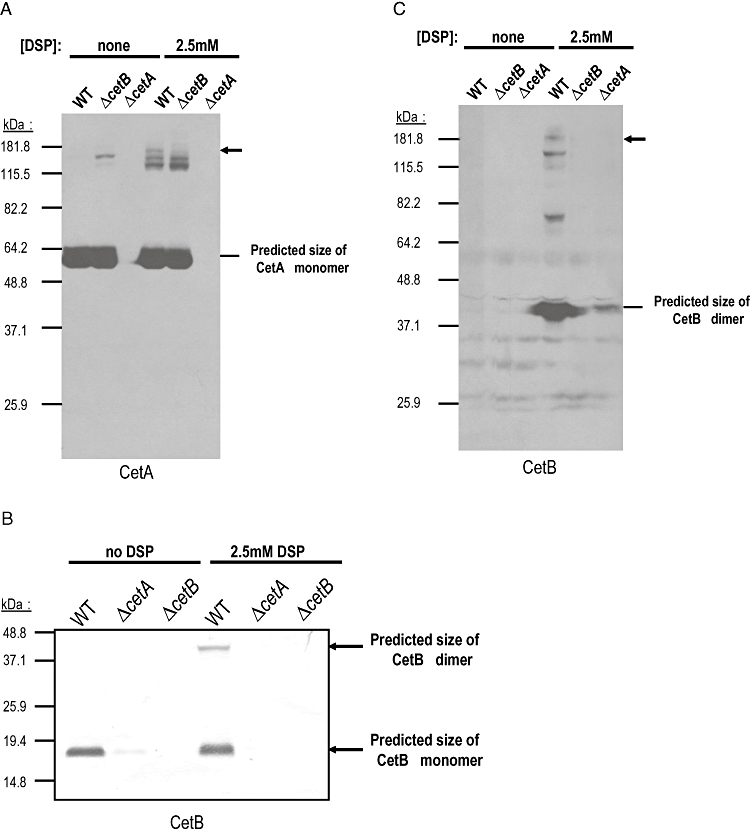
Cross-linking of CetA and CetB. A. Anti-CetA Western blots of DSP cross-linked samples run on 10% SDS-PAGE. Arrow indicates a high molecular weight species present in the wild-type cross-linked sample, but not in the Δ*cetB* cross-linked sample. B. Anti-CetB Western blot of DSP cross-linked lysates run on 12.5% SDS-PAGE. C. Anti-CetB Western blots of DSP cross-linked samples run on 10% SDS-PAGE. Arrow indicates a high molecular weight species of approximately the same size as that in (B). In (A), (B) and (C), wild type, Δ*cetB* and Δ*cetA* cells were treated with either 2.5 mM DSP in DMSO or DMSO alone. The cross-linker (DSP) was quenched and the samples separated by SDS-PAGE (without β-mercaptoethanol or DTT added to the sample buffer) then probed for the presence of CetA and CetB by immunoblotting. In (C), much more protein was loaded than in (B) and the gels were run longer to allow better separation of larger molecular weight species.

When cross-linked wild-type samples were separated on 10% SDS-PAGE and immunoblotted with anti-CetA, several species with molecular weights between approximately 115.5 and 181.8 kDa were apparent (Fig. 7A). The largest of these species (arrow, Fig. 7A) was absent in the cross-linked sample from the Δ*cetB* strain. Another CetA-containing species migrating between approximately 115.5 kDa and 181.8 kDa was present in the Δ*cetB* mutant sample not treated with DSP, but is quite faint in the wild-type sample (Fig. 7A). Upon longer exposure, a band of identical mobility is clearly visible in the wild-type sample (data not shown).

When cross-linked wild-type samples were analysed on 12.5% SDS-PAGE, we detected two species on immunoblotting with anti-CetB (Fig. 7B). One was the predicted size of the CetB monomer (19.3 kDa) and the other was the predicted size of a CetB homodimer (38.6 kDa). In order to detect higher molecular weight CetB complexes, larger amounts of wild-type cross-linked samples were analysed on 10% SDS-PAGE and probed with anti-CetB (Fig. 7C). To obtain separation of larger complexes, the gel was run for a longer period of time, leading the predicted CetB monomer to migrate off the gel. In this Western blot, the apparent homodimer was present, as were several species with higher molecular weights, including one between approximately 64.2 and 82.2 kDa and two major species between approximately 115.5 kDa and 181.8 kDa (Fig. 7C). The largest of these species (arrow, Fig. 7C) was approximately the same size as the largest CetA species (arrow, Fig. 7A).

The identity of each of these CetA and CetB complexes has not been definitively determined, but some inferences can be made. In particular, the largest CetA and CetB complexes are approximately the same size, consistent with a single complex containing both proteins. The molecular weight of this species is between approximately 115.5 and 181.8 kDa, which would be consistent with a complex comprised of two CetA monomers (51.0 kDa each) and two CetB monomers (19.3 kDa each). We also expect that CetA forms a homodimer, as do other MCPs, and this could be one of the CetB-independent species present in the blot shown in Fig. 7A. Further, we expect CetA to interact with other elements of the chemotactic machinery.

## Discussion

**Fig. 8:**
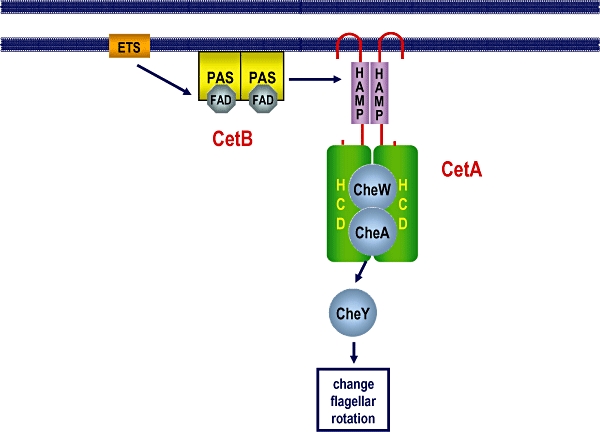
Current model of CetA and CetB localization, topology and function. CetB, which exists as a dimer, is peripherally associated with the membrane, possibly by protein–protein interactions. CetA is an integral membrane protein with two transmembrane domains in a helical hairpin. Our data are consistent with a CetB–CetA interaction, providing a mechanism for transduction of the energy taxis signal to the chemotactic machinery (See *Discussion* for more detail.)

In this study, we carried out molecular and biochemical characterization of CetA and CetB, a putative bipartite energy taxis system of *C. jejuni*. The data we present support a model in which we hypothesize that a membrane-associated CetB dimer serves as a signal-sensing protein and transmits that signal to the integral membrane protein CetA via a direction interaction (Fig. 8). This signal is then transduced to the chemotactic machinery, allowing a change in direction of motility based on changes in the local environment. By analogy with Aer, we hypothesize that the signal recognized by CetB might involve changes in electron transport.

CetA and CetB are encoded by adjacent genes 17 bp apart on the *C. jejuni* chromosome. Based on the small intergenic distance, as well as the fact that both are required for energy taxis (Hendrixson *et al*., 2001), we expected that *cetA* and *cetB* would be co-transcribed. RT-PCR analysis indicated that this is the case (Fig. 1). Additionally, wild-type levels of CetA and CetB were expressed in mutants lacking the sigma factors required for expression of genes involved in flagellar assembly and function, σ^54^ and σ^28^ (Hendrixson and DiRita, 2003) (Fig. 2A). This indicates that neither of these sigma factors is required for expression of the *cetAB* transcript. This differs from *E. coli* and *Salmonella typhimurium*, where *aer* expression appears to be σ^28^-dependent (Park *et al*., 2001; Frye *et al*., 2006). We do not know whether other MCPs are expressed independently of the flagellar regulon in *C. jejuni* or whether *cetA* and *cetB* will prove to be unique in this respect.

Topology prediction programmes indicated that CetA contains a single, fairly long transmembrane helix, and that CetB is entirely cytoplasmic (Fig. 3A and B). Closer examination of the predicted transmembrane helix of CetA led us to hypothesize that this protein instead contains two transmembrane helices separated by a short periplasmic loop, a so-called helical hairpin. We based this prediction on the presence of a histidine, His-24, in the middle of this predicted transmembrane helix. Histidine has been shown to induce helical hairpin formation within transmembrane helices (Monne *et al*., 1999a,b). We tested these topological predictions by making alkaline phosphatase and β-galactosidase fusions at multiple locations within CetA, including His-24 (Fig. 4A). The enzyme activities of these fusions are consistent with CetA containing two transmembrane regions and a short periplasmic loop that includes His-24 (Fig. 4B and C). Further studies are necessary to precisely define the ends of this loop. However, there are two glycines present 4–5 residues N-terminal and C-terminal to His-24. Glycine also displays some turn-inducing tendencies in transmembrane helices and appears to predominate as the N-terminal residue in helical hairpin loops (Monne *et al*., 1999b). Accordingly, we would predict that the periplasmic loop of CetA may extend from Gly-19 to His-24, potentially continuing on to Gly-28. From our topology analysis with CetA, we arrive at a conclusion similar to the Aer topology model, which was derived biochemically (Amin *et al*., 2006).

The sequence analysis and fusion data indicate that CetA is an integral membrane protein and CetB is cytoplasmic. We further examined the subcellular location of both CetA and CetB when expressed from the chromosome of *C. jejuni*. These experiments demonstrated that both CetA and CetB localize to the membrane of *C. jejuni* (Fig. 5). Membrane extraction experiments, using various conditions to disrupt protein associations with the membrane, indicated that CetA is an integral membrane protein and CetB is a peripheral membrane protein (Fig. 6). As CetB is partially released from the membrane following urea treatment, but not high-salt treatment, we conclude that CetB is membrane-associated via a strong interaction, likely with other proteins as opposed to the membrane itself. We predict that CetB associates with the membrane by interacting with either CetA or an unidentified element(s) of the ETS whose redox state CetB is predicted to sense. Attempts to determine whether or not the membrane association of CetB is CetA-dependent have been confounded by the extremely low level of CetB expression in the absence of CetA.

Based on the fact that CetA and CetB possess all of the domains of the energy taxis receptor Aer and are required for energy taxis by *C. jejuni* (Hendrixson *et al*., 2001), we predict that CetA and CetB interact directly to transduce an energy taxis signal. We observed that CetB levels are extremely low in the absence of CetA, whether CetB is expressed from the chromosome or from a multicopy plasmid (Fig. 2). Protein–protein interactions often manifest themselves in the loss of stability of one protein when the other is not present. This may be the case with CetA and CetB. However, at present we cannot rule out that CetA may instead have an effect on the levels of translation of CetB. If the latter scenario is true, then the *cis* element via which CetA affects translation must be strictly contained within the coding sequence of *cetB*, based on the maintenance of the CetA-dependent effect when the *cetB* orf is expressed constitutively from a multicopy plasmid. One could differentiate between an effect of CetA on stability or on translation of CetB using pulse-chase experiments. However, such experiments would require either an inducible promoter system, which has not yet been developed for *C. jejuni*, or the ability to immunoprecipiate CetB, which we have been unable to do despite repeated attempts.

Evidence of an interaction between CetA and CetB was obtained from *in vivo* cross-linking experiments. In these experiments, whole cells were treated with the membrane permeable primary amine cross-linker DSP. When the cross-linked samples were probed for the presence of CetA by Western blot, a high molecular weight species was evident in the wild-type sample that was absent in the Δ*cetB* mutant sample (Fig. 7A). A high molecular weight species of similar size was also observed by anti-CetB Western blot (Fig. 7C). Owing to the low levels of CetB in the Δ*cetA* mutant, we cannot be conclusive as to whether this species is present in the Δ*cetA* mutant sample. The size of this cross-linked species is consistent with a complex consisting of two CetA monomers and two CetB monomers. These data, together with the fact that CetB levels are CetA-dependent, are consistent with an interaction between CetA and CetB. Efforts to gather more direct evidence for such an interaction by yeast two-hybrid (Parrish *et al.*, 2007) and bacterial two-hybrid systems (data not shown) have been unsuccessful. Membrane proteins have often proved difficult to analyse using these methods. Additionally, CetB appears to form inclusion bodies when expressed at high levels in *E. coli* (data not shown), further confounding this approach and others involving expression in this background. Our results are consistent with an interaction between CetA and CetB, although further studies are necessary to develop more direct evidence for or against such an interaction.

The HAMP domains of CetA and Aer differ significantly (K.T. Elliott, I.B. Zhulin, J.A. Stuckey, V.J. DiRita, in revision). Based on similarity to the HAMP domain of CetA, we identified a family of 55 pairs of CetA- and CetB-like proteins (which we call HAMP/PAS pairs) in a diverse group of bacterial species (K.T. Elliott, I.B. Zhulin, J.A. Stuckey, V.J. DiRita, in revision). The HAMP domains of this family contain nine conserved residues which we suggest may define a PAS–domain interaction surface. Single alanine substitutions at these positions do not alter the localization of CetB to the membrane. If these conserved residues are involved in CetA–CetB interactions, we predict that substitutions at multiple positions within this region may lead to a change in CetB stability, CetB membrane localization and/or CetA–CetB complex formation.

Our *in vivo* cross-linking experiments also indicate that CetB forms a homodimer (Fig. 7B). PAS domains often form dimers, but it was not previously known if this was the case with CetB. In addition, whether or not the PAS domain of Aer dimerizes has yet to be established (Taylor, 2007). Further experiments aimed at defining the dimerization surface of CetB may provide genetic evidence for or against dimerization of the Aer PAS domain, based on similarity or dissimilarity in this region. The identification of CetB mutants deficient in dimerization would also allow us to test whether CetB dimer formation is required for CetA/CetB-directed behaviour. The *in vivo* cross-linking experiments also demonstrated that CetA and CetB participate in several other high molecular weight complexes. Such complexes are expected, as interactions with the chemotaxis machinery would be necessary for CetA and CetB to transduce a taxis signal.

We can make some predictions about CetB based on sequence comparisons with the PAS domain of Aer. Three residues in the PAS domain of Aer (Arg-57, His-58 and Asp-60) are required for FAD binding (Repik *et al*., 2000), are located close to the predicted FAD binding site and are conserved in Aer-like (FAD-binding) PAS domains (personal communication in Taylor, 2007). These residues align with identical or similar residues in CetB (Arg-50, His-51 and Glu-53). Based on these similarities, we predict that CetB binds an FAD cofactor. Additionally, the HAMP domain of Aer is required for proper folding and FAD binding by the PAS domain. This requirement, however, can be subverted by non-specific suppressor mutations in the PAS domain (S28G, A65V and A99V) which allow FAD binding in the presence of HAMP domain point mutations that usually abrogate FAD binding (Watts *et al*., 2004; Buron-Barral *et al*., 2006). These suppressing residues (Gly-28, Val-65 and Val-99) are the naturally occurring residues at the equivalent positions in CetB (Gly-21, Val-58 and Val-92). This intriguing fact suggests the hypothesis that CetB may fold and bind FAD without interacting with the CetA HAMP domain.

These studies have allowed us to further refine our model of energy taxis signal transduction by CetA and CetB (Fig. 8) and make testable predictions about how this bipartite system works vis-à-vis what is known about the Aer single protein energy taxis receptor. The molecular nature of this interaction likely differs significantly from that within Aer (K.T. Elliott, I.B. Zhulin, J.A. Stuckey, V.J. DiRita, in revision). The work presented here has provided information on the expression and biochemical properties of CetA and CetB, allowing us to further our understanding of these proteins and to build a foundation for future studies aimed at elucidating the molecular mechanisms of signal transduction within this putative energy taxis system.

## Experimental procedures

### Bacterial strains and culture conditions

All bacterial strains and plasmids used in this study are listed in Table 1. DRH212, a spontaneous streptomycin-resistant mutant of the clinical isolate *C. jejuni* 81–176, was the background strain for all mutants studied and is referred to as wild type (Hendrixson *et al*., 2001). *C. jejuni* was routinely grown on Mueller–Hinton (MH) agar with 10 μg ml^−1^ trimethoprim under microaerobic conditions (85% N_2_, 10% CO_2_, 5% O_2_) in a tri-gas incubator. Biphasic cultures were grown in 20 ml MH broth overlaid on 20 ml MH agar under microaerobic conditions. For *C. jejuni*, the following antibiotic concentrations were used: 10 μg ml^−1^ trimethoprim, 30 μg ml^−1^ cefaperazone, 50 μg ml^−1^ kanamycin, 20 μg ml^−1^ chloramphenicol and 0.1 or 2 mg ml^−1^ streptomycin. *E. coli* was grown in Luria–Bertani (LB) agar or broth. For *E. coli*, the following antibiotic concentrations were used: 50 μg ml^−1^ kanamycin, 30 μg ml^−1^ chloramphenicol or 100 μg ml^−1^ ampicillin.

**Table 1:** Bacterial strains and plasmids.

Strain or plasmid	Relevant characteristics	Reference
Bacteria		
*E. coli*		
JM101	F′*traD36 proA*^+^*B*^+^*lacI*^*q*^Δ*(lacZ)M15/*Δ*(lac-proAB) glnV thi*	New England Biolabs
DH5α/pRK212.1	contains conjugative plasmid for transfer of plasmid into *C. jejuni*	Figurski and Helinski (1979)
TG1	Δ*hsdS Δ(lac-proAB) supE thi F*′*[traD36 proAB*^+^*lacl*^*q*^*ΔlacZM15]*	Sambrook *et al*. (1989)
*C. jejun*		
DRH212	81–176 Sm, spontaneous mutant	Hendrixson *et al*. (2001)
DRH307	Δ*cetB*	Hendrixson *et al*. (2001)
DRH311	Δ*fliA*	Hendrixson *et al*. (2001)
DRH321	Δ*rpoN*	Hendrixson *et al*. (2001)
DRH333	Δ*cetA*	Hendrixson *et al*. (2001)
KYCj172	Δ*cetAB*	This study
Plasmids		
pRY108	Km^R^, *E. coli*/*C. jejuni* shuttle vector	Yao *et al*. (1993)
pECO102	*C. jejuni* plasmid for gene expression from cat promoter, Cm^R^	Wiesner *et al*. (2003)
pECO101	pRY108 derivative with *cat* promoter in XhoI*-*BamHI site, Km^R^	This study
pTrcphoA	pTrc99a containing ‘*phoA*’ (lacks *phoA* codons 1–26)	Blank and Donnenberg (2001)
pTrclacZ	pTrc99a containing ‘*lacZ*’ (lacks *lacZ* codons 1–7)	Blank and Donnenberg (2001)
pKTY360	pRY108 with 2.4 kb fragment containing *cetA* and *cetB* coding sequence cloned into the XmnI site	This study
pKTY367	pKTY360 with the Y116A mutation in the *cetA* coding sequence	This study
pKTY213	pECO101 with *cetB* cloned into the BamHI and XhoI sites	This study
pKTY300	pECO101 with *cetAB* cloned into BamHI and XhoI sites	This study
pKTY333	pTrcphoA with *cetA* codons 1–5 cloned into NcoI and XmaI sites	This study
pKTY349	pTrcphoA with *cetA* codons 1–24 cloned into NcoI and XmaI sites	This study
pKTY344	pTrcphoA with *cetA* codons 1–50 cloned into NcoI and XmaI sites	This study
pKTY343	pTrcphoA with *cetA* codons 1–140 cloned into NcoI and XmaI sites	This study
pKTY334	pTrcphoA with *cetA* (full length) cloned into NcoI and XmaI sites	This study
pKTY332	pTrclacZ with *cetA* codons 1–5 cloned into NcoI and XmaI sites	This study
pKTY328	pTrclacZ with *cetA* codons 1–24 cloned into NcoI and XmaI sites	This study
pKTY327	pTrclacZ with *cetA* codons 1–50 cloned into NcoI and XmaI sites	This study
pKTY329	pTrclacZ with *cetA* codons 1–140 cloned into NcoI and XmaI sites	This study
pKTY326	pTrclacZ with *cetA* (full length) cloned into NcoI and XmaI sites	This study
pBW208	pECO102 with *ctsP* and C-terminal FLAG tag	Lab stock

### Construction of *ΔcetAB* deletion mutant

The Δ*cetAB* deletion mutant was constructed essentially as described by Hendrixson *et al*. (2001). The *cetA* and *cetB* coding sequences with 1036 bp upstream and 595 bp downstream were amplified by PCR with primers designed with KpnI sites at their 5′ ends for cloning into pUC19. The resulting plasmid was pKTY60. A deletion from the first codon of *cetA* to the last codon of *cetB* was created via Pfu mutagenesis (Weiner *et al*., 1994). This plasmid, pKTY62, was electroporated into DRH304, which harbours the *cat-rpsL* cassette in the *cetB* coding sequence. Transformants were selected on 2 mg ml^−1^ streptomycin and screened for sensitivity on 20 μg ml^−1^ chloramphenicol. The deletion was confirmed by PCR analysis and chromosomal sequencing.

### Construction of plasmids for gene expression in *C. jejuni*

To construct a kanamycin selectable plasmid for gene expression in *C. jejuni*, an 82 bp fragment containing the promoter for the *C. jejuni* chloramphenicol acetyltransferase (*cat*) gene from pRY109 (Yao *et al*., 1993) was amplified by PCR using primers containing 5′ XbaI and BamHI sites*.* These primers were used to amplify the 82 bp fragment, and the resulting fragment cloned into pRY108 (Yao *et al*., 1993) giving rise to the plasmid pECO101. Except for antibiotic selection, pECO101 functions similarly to the previously constructed plasmid pECO102 (Wiesner *et al*., 2003). To construct a plasmid expressing *cetB* from the *cat* promoter, the *cetB* coding sequence was amplified by PCR with primers containing restriction sites so that a BamHI site was added immediately 5′ to *cetB* and an XhoI site immediately 3′ to *cetB* for cloning into pECO101. To construct a plasmid expressing both *cetA* and *cetB* from the *cat* promoter, the *cetA* and *cetB* coding sequences and intergenic region were amplified by PCR with primers containing restriction sites so that a BclI site was added immediately 5′ to *cetA* and an XhoI site immediately 3′ to *cetB*. The resulting fragment was digested with BclI and XhoI and cloned into the BamHI and XhoI sites of pECO101. All plasmids were confirmed by DNA sequencing.

### Construction of a plasmid to complement the *ΔcetAB* mutant

pKTY60 was digested with ApaLI and BsrBI. The resulting fragment containing the *cetA* and *cetB* coding sequences, along with 299 bases upstream and 202 bases downstream, was blunted by T4 DNA polymerase. This fragment was then cloned into the XmnI site in the *E. coli*/*C. jejuni* shuttle vector pRY108 (Yao *et al*., 1993). The resulting plasmid, pKTY360, lacks the 5′ 325 bp of the *cj1191c* open-reading frame.

### Site-directed mutagenesis

Mutation of the *cetA* coding sequence leading to an alanine substitution at residue 116 (Y116A) was made in pKTY60 using Pfu mutagenesis (Weiner *et al*., 1994). DNA sequence of the resulting plasmid was determined to confirm the presence of the point mutation and ensure the absence of additional mutations. This plasmid was then digested with ApaLI and BsrBI and the resulting fragment cloned into the XmnI site of pRY108 as described above. The orientation of the insertions into pRY108 was checked by multiple restriction digests to confirm that the resulting plasmid is identical to pKTY360 except for the indicated point mutations.

### Conjugation of plasmids into *C. jejuni*

Plasmids were conjugated into *C. jejuni* as described by Guerry *et al*. (1994). Briefly, *C. jejuni* was grown on MH agar with 10 μg ml^−1^ trimethoprim for 16–20 h and re-suspended in MH broth to an OD_600_ of 1.0. Overnight cultures of the *E. coli* donor strain [DH5α(pRK212.1) containing the plasmid to be conjugated into *C. jejuni*] were diluted into fresh LB broth and grown to an OD_600_ of 0.5. A total of 500 μl of the donor culture was centrifuged and the pellet washed twice with MH broth, then re-suspended in 1 ml of the *C. jejuni* recipient culture. This mixture was spotted onto MH agar with no antibiotics. After 5 h at 37°C in microaerobic conditions, the bacteria were re-suspended and spread onto MH agar containing 10 μg ml^−1^ trimethoprim, 30 μg ml^−1^ cefaperazone, 2 mg ml^−1^ streptomycin and 50 μg ml^−1^ kanamycin. PCR was used to verify transfer of the plasmid to the recipient *C. jejuni* strain.

### RNA extractions and RT-PCR

*Campylobacter jejuni* strains were grown in biphasic cultures for 48 h. RNA extractions were performed using Qiagen RNAprotect and Qiagen RNeasy according to manufacturer's instructions, without the use of on-column DNase treatment. To eliminate contaminating DNA, 10× DNase buffer (200 mM sodium acetate pH 4.5, 100 mM MgCl_2_, 100 mM NaCl) and 10 units of DNase I (RNase-free, Roche) were added to each RNA sample and incubated at room temperature for 1 h. DNase was removed by sequential phenol and chloroform extractions, followed by ethanol precipitation. The final concentration of RNA in each sample was quantified by OD_260_.

Qualitative RT-PCR was performed as follows. A total of 2.5 μg of RNA was mixed with 3 μg random primers (Invitrogen) and cDNA synthesized using MMLV reverse transcriptase (Invitrogen) according to manufacturer's instructions. Control reactions with MMLV reverse transcriptase omitted were performed simultaneously to detect any contaminating DNA. Equal amounts of cDNA products were then used as a template for PCR using either two primers within *rpoA* or one primer within *cetA* and one primer within *cetB*. Control reactions using genomic DNA as a template were also performed. RT-PCR products were separated on a 0.8% agarose gel and visualized with ethidium bromide.

### SDS-PAGE and Western blots

For SDS-PAGE of whole cell lysates, *C. jejuni* strains were grown on MH agar for 16–20 h, then re-suspended in MH broth to an OD_600_ of 0.8. The bacteria were pelleted by centrifugation and the pellet re-suspended in 100 μl 2× sample buffer. All other samples were normalized by protein concentration or OD_600_ as indicated below. Samples were boiled then separated on 10% or 12.5% polyacrylamide gels (as indicated). Proteins were transferred to nitrocellulose membranes and probed with rabbit anti-CetA (1:10 000–1:75 000, generated against an internal peptide by Open Biosystems) or rabbit anti-CetB (1:500–1:5000, generated against an internal peptide by Open Biosystems) followed by either goat anti-rabbit alkaline phosphatase-conjugated secondary antibody (1:10 000, Zymed) or goat anti-rabbit peroxidase-conjugated secondary antibody (1:20 000, Pierce). For detection of CtsP-FLAG, membranes were probed with anti-FLAG peroxidase-conjugated antibody (1:1000, Sigma). Alkaline phosphatase probed Western blots were developed using the chromogenic substrate 5-bromo-4-chloro-3-indolyl phosphate/nitro blue tetrazolium as previously described (Sambrook *et al*., 1989). Peroxidase-probed Western blots were developed using the Western Lightning kit (PerkinElmer).

### Topology predictions

Transmembrane domain predictions were preformed using DAS (http://www.sbc.su.se/~miklos/DAS). Signal sequence predictions were performed using LipoP (http://www.cbs.dtu.dk/services/LipoP). Hydrophobicity of individual residues within the predicted CetA transmembrane region was assessed by plotting the Kyte–Doolittle value of each residue in this region. This approach resembles that used recently to analyse the attributes of individual HAMP domain residues (Hulko *et al*., 2006) and differs from the usual Kyte–Doolittle analysis, which gives the average hydrophobicity of 19 residues centred at each position.

### Topology analysis using PhoA and LacZ fusions

The plasmids pTrcphoA and pTrclacZ were used to construct plasmids containing C-terminal PhoA or LacZ fusions to full-length or truncated CetA. pTrcphoA consists of the coding sequence for *phoA* without codons 1–26 (the signal sequence), denoted *‘phoA*, cloned into pTrc99a’ (Blank and Donnenberg, 2001). pTrclacZ consists of the coding sequence of *lacZ* without codons 1–7, denoted ‘*lacZ*, cloned into pTrc99a’ (Blank and Donnenberg, 2001). Truncations of *cetA* consisting of the first 24, first 50 or first 140 codons and full-length *cetA* were amplified using primers that added an NcoI site immediately 5′ and an XmaI site immediately 3′ to the coding sequence for cloning into the NcoI and XmaI sites of pTrcphoA and pTrclacZ. The first five codons of *cetA* were inserted between the NcoI and XmaI sites of pTrcphoA and pTrclacZ using Pfu mutagenesis (Weiner *et al*., 1994). All resulting plasmids were confirmed by DNA sequencing.

Each of the above plamids, including the original vectors, was transformed into *E. coli* strain TG1, which lacks *phoA* and *lacZ*. Alkaline phosphatase and β-galactosidase activities of each strain were assessed as previously described (Miller, 1972; Manoil, 1991). Assays were performed in triplicate and the average and standard deviation calculated for each strain.

### Subcellular fractionation

*Campylobacter jejuni* strains were grown on MH agar for 16–20 h and re-suspended in 10 mM HEPES pH 7.4. Cells were lysed by one freeze-thaw cycle, followed by sonication 3–6 times for 10 s. Cellular debris was removed by centrifugation at 10 000 *g* for 10 min. Soluble and membrane fractions were separated by ultracentrifugation at 100 000 *g* for 1 h. Following ultracentrifugation, the supernatant contained soluble (cytoplasmic and periplasmic proteins) and the pellet contained insoluble (membrane) proteins. Protein concentrations were quantified using the Bio-Rad Protein Assay. Equal amounts of protein from each sample were run on SDS-PAGE for Western analysis.

### Isocitrate dehydrogenase assays

Subcellular fractions were assayed for isocitrate dehydrogenase activity as previously described (Myers and Kelly, 2005). Briefly, equal amounts of protein from each fraction were incubated with 5 mM Tris pH 8.0, 1 mM nicotinamide adenine dinucleotide phosphate (NADP), 1 mM MgCl_2_ and 5 mM sodium isocitrate at room temperature. Isocitrate dehydrogenase activity was monitored by measuring the rate of increase of OD_340_, which represents the rate of NADPH production. The per cent of isocitrate dehydrogenase specific activity within each fraction was determined. For all fractionation experiments, at least 90% of the isocitrate dehydrogenase specific activity was found in the soluble fraction.

### Membrane extraction

*Campylobacter jejuni* strains were grown and fractionated into soluble and membrane fractions as described above. The membrane fraction was then re-suspended in 10 mM HEPES pH 7.4, incubated at 4°C with rocking for 30 min to 1 h, followed by ultracentrifugation at 100 000 *g* for 1 h. This wash step was repeated three times. The washed membrane fraction was then mixed 1:1 with 10 mM HEPES pH 7.4 or 10 mM HEPES pH 7.4 containing 3 M urea, 5 M urea, 12 M urea, 1 M NaCl or 3 M NaCl (concentrations given are twice the final concentration). These mixtures were incubated at 4°C with rocking for 30 min to 1 h, followed by ultracentrifugation at 100 000 *g* for 1 h. Following ultracentrifugation, the supernatant contained soluble proteins and the pellet insoluble proteins. The soluble proteins were precipitated with cold acetone. Both soluble and insoluble samples were re-suspended in an equal volume of 10 mM HEPES pH 7.4 and mixed 1:1 with 2× sample buffer. Equal volumes of each sample were used for SDS-PAGE and Western analysis.

### *In vivo* cross-linking

*Campylobacter jejuni* strains were grown on MH agar for 16–20 h then re-suspended in MH broth to an OD_600_ of 8.0. A total of 2.5 mM dithiobis(succinimidyl)propionate (DSP) in DMSO was added to each culture, with additional DMSO added to bring the combined DSP and DMSO volume to 1/10th of the final volume. Untreated samples received 1/10th final volume of DMSO. Samples were incubated at room temperature in ambient atmosphere for 20 min 50 mM Tris pH 8.0 was added to each sample to quench any remaining DSP. Equal volumes of each samples were run on SDS-PAGE without β-mercaptoethanol or DTT added to the sample buffer, as these would cleave the DSP mediated cross-linking.
